# Real-world study of overall survival with palbociclib plus aromatase inhibitor in HR+/HER2− metastatic breast cancer

**DOI:** 10.1038/s41523-022-00479-x

**Published:** 2022-10-11

**Authors:** Hope S. Rugo, Adam Brufsky, Xianchen Liu, Benjamin Li, Lynn McRoy, Connie Chen, Rachel M. Layman, Massimo Cristofanilli, Mylin A. Torres, Giuseppe Curigliano, Richard S. Finn, Angela DeMichele

**Affiliations:** 1 University of California San Francisco Helen Diller Family Comprehensive Cancer Center, San Francisco, CA USA; 2 UPMC Hillman Cancer Center, University of Pittsburgh Medical Center, Pittsburgh, PA USA; 3 Pfizer Inc, New York, NY USA; 4 The University of Texas MD Anderson Cancer Center, Houston, TX USA; 5 Weill Cornell Medicine, New York, NY USA; 6 Winship Cancer Institute, Emory University School of Medicine, Atlanta, GA USA; 7 European Institute of Oncology, IRCCS and University of Milano, Milan, Italy; 8 David Geffen School of Medicine at University of California Los Angeles, Santa Monica, CA USA; 9 Abramson Cancer Center, University of Pennsylvania, Philadelphia, PA USA

**Keywords:** Breast cancer, Targeted therapies

## Abstract

Data on real-world effectiveness of cyclin-dependent kinase 4/6 inhibitor combination therapy versus endocrine therapy alone are limited. The Flatiron Health Analytic Database was used to assess overall survival (OS) in patients with hormone receptor–positive/human epidermal growth factor receptor 2–negative (HR+/HER2−) metastatic breast cancer (MBC) treated with first-line palbociclib plus an aromatase inhibitor (AI) versus an AI alone in routine US clinical practice. In total, 2888 patients initiated treatment during February 3, 2015–March 31, 2020, with a potential ≥6-month follow-up (cutoff date, September 30, 2020). After stabilized inverse probability treatment weighting, median OS (95% CI) is significantly longer among palbociclib versus AI recipients (49.1 [45.2–57.7] versus 43.2 [37.6–48.0] months; hazard ratio, 0.76 [95% CI, 0.65–0.87]; *P* < 0.0001). Progression-free survival (95% CI) is 19.3 (17.5–20.7) versus 13.9 (12.5–15.2) months, respectively (hazard ratio, 0.70 [95% CI, 0.62–0.78]; *P* < 0.0001). These data support first-line palbociclib plus an AI treatment for HR+/HER2− MBC.

(Trial number NCT05361655).

## Background

Breast cancer accounts for nearly one-third of all cancer cases among women^1^. In the United States in 2022, approximately 290,560 new cases of breast cancer will be diagnosed, 287,850 among women and 2710 among men, with an estimated 43,250 and 530 deaths, respectively. About 6% of breast cancers are diagnosed as metastatic breast cancer (MBC) indicating that the cancer has spread to distant tissues. The 5-year survival rate for de novo MBC is only 29.0%^2^.

The majority (68%) of breast cancer cases have a hormone receptor–positive (HR+)/human epidermal growth factor receptor 2–negative (HER2−) subtype. As first-line treatment for pre- and postmenopausal women and for men with HR+/HER2− MBC, the NCCN Clinical Practice Guidelines in Oncology (NCCN Guidelines®) recommend a cyclin-dependent kinase 4/6 (CDK4/6) inhibitor in combination with endocrine therapy^3^. The CDK4/6 inhibitor palbociclib was approved in February 2015 as first-line treatment for HR+/HER2− MBC in combination with an aromatase inhibitor (AI) and was approved in February 2016 in combination with fulvestrant for patients who progressed while receiving prior endocrine therapy^4–6^. The palbociclib label was also expanded in 2019 to include men with HR+/HER2− MBC^7^. In the phase 3 PALOMA-2 trial, first-line palbociclib plus letrozole versus letrozole plus placebo significantly prolonged median progression-free survival (PFS) in women with estrogen receptor–positive/HER2− MBC^8,9^, although median overall survival (OS), a secondary endpoint, was numerically higher among patients who received palbociclib plus letrozole versus letrozole plus placebo, but the difference was not statistically significant (53.9 vs 51.2 months, *P* > 0.05)^10^.

Real-world evidence can be used to understand the effectiveness of a drug in routine clinical practice and, through the inclusion of patients who may be underrepresented in clinical trials, may help to inform the treatment of patients in routine care^11–13^. A recent systematic literature review summarized real-world studies of a CDK4/6 inhibitor as treatment for HR+/HER2− MBC and showed that real-world data were consistent with clinical trial findings and that CDK4/6 inhibitors are safe and effective treatments for HR+/HER2− MBC in routine practice^14^. Of note, palbociclib was the predominant CDK4/6 inhibitor assessed in those real-world studies.

The interpretation of real-world studies may be limited by the lack of a comparator group, small sample size, short follow-up, and/or differences in outcome endpoint definitions^15–18^. Only a few comparative effectiveness analyses of CDK4/6 inhibitor outcomes in MBC have been published to date, including DeMichele et al. (2021) and Brufsky et al. (2021) using the Flatiron Health Analytic Database (Flatiron Health, New York, NY) and Ha et al. (2022) from one academic institution (Breast Medical Oncology database; MD Anderson Cancer Center, Houston, TX)^19–21^. Using the Flatiron Database, a comparative effectiveness real-world analysis demonstrated longer real-world PFS (rwPFS) and OS among all patients treated with palbociclib plus letrozole versus letrozole alone^20^ and among patients with at least one tumor response assessment^19^. These analyses had a relatively small sample size and short follow-up time and were comparative with letrozole only. Therefore, additional research with both men and women, with an AI as the endocrine partner as per the palbociclib label and with longer-term follow-up, is warranted to further evaluate these outcome findings in the real-world setting.

This real-world analysis (P-REALITY X: Palbociclib REAl-world first-LIne comparaTive effectiveness studY eXtended) uses the Flatiron Database to evaluate OS and rwPFS of palbociclib plus an AI versus an AI alone in postmenopausal women and in men with HR+/HER− MBC in routine clinical practice in the United States with a follow-up time up to 68 months from the index date to data cutoff date.

## Results

Interactive visualization of the data presented in this article is available at: https://realworld-data.dimensions.ai/p-reality-x.

### Patients

**Fig. 1:**
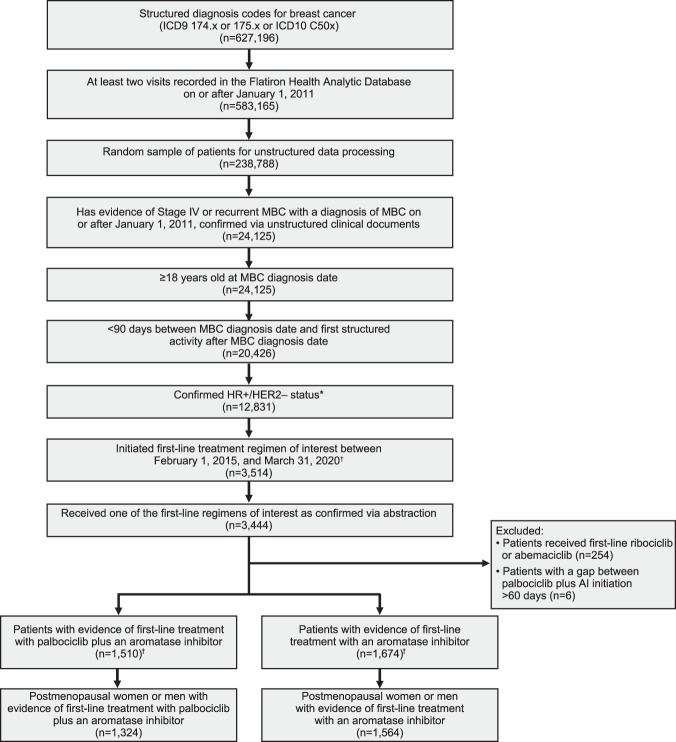
Patient attrition diagram. ER+  estrogen receptor–positive; HER2−  human epidermal growth factor receptor 2–negative; HR+  hormone receptor–positive; ICD9/10 *International Classification of Diseases, 9th/10th Revision*; MBC  metastatic breast cancer; PR+  progesterone receptor–positive. *Confirmed HR+/HER2− status: HR+ is defined as any ER+ or PR+ biomarker test before or up to 60 days after metastatic diagnosis; HER2− is defined as any HER2− test and the absence of a positive test before or up to 60 days after metastatic diagnosis. ^†^Lines were selected regardless of whether they contained a luteinizing hormone–releasing hormone agonist (leuprolide, goserelin, and triptorelin).

From February 3, 2015, to March 31, 2020, a total of 2888 postmenopausal women and men with HR+/HER2− MBC from the Flatiron Database started treatment with palbociclib plus an AI (*n* = 1324) or with an AI alone (*n* = 1564) as first-line therapy (Fig. 1). Ten men were included in the palbociclib group and 19 men in the AI alone group (Table 1). Most patients (>90%) were treated in the community versus academic setting, and the percentage of patients with different insurance plans was similar between treatment groups. More patients treated with palbociclib plus an AI had an Eastern Cooperative Oncology Group (ECOG) performance status of 0, de novo MBC, a lower mean comorbidity score, and a higher number of metastatic sites compared with patients who received an AI alone (Table 2). Patient characteristics were generally balanced after stabilized inverse probability treatment weighting (sIPTW) and between propensity score-matched groups. After sIPTW, the median age was 70 years in both treatment groups. The majority of patients (~68%) were White in each treatment group, and about 30% of patients had visceral disease. After sIPTW, the median duration of follow-up was 23.9 months (interquartile range, 12.8–38.0) in the palbociclib plus an AI group and 24.5 months (12.0–42.9) in the AI alone group.

**Table 1:** Patient demographic characteristics.

	Unadjusted cohort			Cohort after stabilized inverse probability treatment weighting			Cohort after propensity score matching		
Characteristic	Palbociclib + aromatase inhibitor (*n* = 1324)	Aromatase alone (*n* = 1564)	Standardized difference	Palbociclib + aromatase (*n* = 1572)	Aromatase alone (*n* = 1137)	Standardized difference	Palbociclib + aromatase (*n* = 939)	Aromatase alone (*n* = 939)	Standardized difference
Age, y									
Mean (SD)	67.1 (9.6)	70.9 (9.7)	−0.3949	69.4 (10.8)	69.5 (8.2)	−0.0161	68.7 (9.5)	69.4 (9.4)	−0.0783
Median (inter- quartile range)	67 (61–74)	72 (64–80)		70 (63–78)	70 (63–79)		69 (63–76)	70 (63–78)	
Age group, *n* (%), y									
18−49	48 (3.6)	41 (2.6)	0.0577	44 (2.8)	34 (3.0)	−0.0134	26 (2.8)	22 (2.3)	0.0270
50–64	468 (35.4)	375 (24.0)	0.2509	437 (27.8)	329 (28.9)	−0.0238	257 (27.4)	269 (28.7)	−0.0285
65–74	495 (37.4)	500 (32.0)	0.1140	532 (33.8)	394 (34.7)	−0.0172	376 (40.0)	356 (37.9)	0.0437
≥75	313 (23.6)	648 (41.4)	−0.3868	559 (35.6)	380 (33.5)	0.0445	280 (29.8)	292 (31.1)	−0.0278
Sex, n (%)									
Male	10 (0.76)	19 (1.2)	−0.0465	17 (1.1)	12 (1.0)	0.0056	8 (0.85)	10 (1.1)	−0.0219
Female	1314 (99.2)	1545 (98.8)		1555 (98.9)	1125 (99.0)		931 (99.2)	929 (98.9)	
Race/ethnicity, *n* (%)									
White	900 (68.0)	1059 (67.7)	0.0057	1063 (67.6)	766 (67.4)	0.0044	591 (62.9)	636 (67.7)	−0.1008
Black	107 (8.1)	136 (8.7)	−0.0222	134 (8.5)	96 (8.5)	0.0019	83 (8.8)	71 (7.6)	0.0466
Other/unknown	317 (23.9)	369 (23.6)	0.0082	375 (23.9)	274 (24.1)	−0.0060	265 (28.2)	232 (24.7)	0.0797
Practice type, *n* (%)									
Community	1208 (91.2)	1449 (92.7)	−0.0518	1449 (92.2)	1048 (92.1)	0.0016	865 (92.1)	868 (92.4)	−0.0120
Academic	116 (8.8)	115 (7.4)		123 (7.8)	89 (7.9)		74 (7.9)	71 (7.6)	
Insurance, *n* (%)									
Commercial health plan plus any other	388 (29.3)	507 (32.4)	−0.0674	474 (30.2)	353 (31.0)	−0.0182	290 (30.9)	292 (31.1)	−0.0046
Commercial health plan	332 (25.1)	325 (20.8)	0.1023	372 (23.7)	251 (22.1)	0.0375	208 (22.2)	210 (22.4)	−0.0051
Medicare	59 (4.5)	72 (4.6)	−0.0071	67 (4.3)	48 (4.3)	0.0011	46 (4.9)	37 (3.9)	0.0466
Medicaid	16 (1.2)	15 (0.96)	0.0241	16 (1.0)	12 (1.0)	−0.0030	9 (0.96)	8 (0.85)	0.0112
Other payer type	529 (40.0)	645 (41.2)	−0.0262	643 (40.9)	473 (41.6)	−0.0148	386 (41.1)	392 (41.8)	−0.0130

**Table 2:** Patient clinical characteristics.

	Unadjusted cohort			Cohort after stabilized inverse probability treatment weighting			Cohort after propensity score matching		
Characteristic	Palbociclib + aromatase inhibitor (*n* = 1324)	Aromatase inhibitor alone (*n* = 1564)	Standardized difference	Palbociclib + Aromatase Inhibitor (*n* = 1572)	Aromatase inhibitor alone (*n* = 1137)	Standardized difference	Palbociclib + aromatase inhibitor (*n* = 939)	Aromatase inhibitor alone (*n* = 939)	Standardized difference
Disease stage at initial diagnosis, *n* (%)									
I	147 (11.1)	216 (13.8)	−0.0821	198 (12.6)	145 (12.8)	−0.0060	114 (12.1)	121 (12.9)	−0.0225
II	345 (26.1)	418 (26.7)	−0.0152	407 (25.9)	300 (26.4)	−0.0118	262 (27.9)	247 (26.3)	0.0359
III	181 (13.7)	297 (19.0)	−0.1443	261 (16.6)	188 (16.6)	0.0011	144 (15.3)	150 (16.0)	−0.0176
IV	541 (40.9)	464 (29.7)	0.2359	530 (33.7)	390 (34.3)	−0.0110	323 (34.4)	323 (34.4)	0.0000
Not documented	110 (8.3)	169 (10.8)	−0.0850	176 (11.2)	114 (10.0)	0.0389	96 (10.2)	98 (10.4)	−0.0070
Eastern Cooperative Oncology Group performance status, *n* (%)									
0	499 (37.7)	397 (25.4)	0.2672	472 (30.1)	348 (30.6)	−0.0126	273 (29.1)	304 (32.4)	−0.0716
1	318 (24.0)	334 (21.4)	0.0636	362 (23.0)	259 (22.8)	0.0066	228 (24.3)	225 (24.0)	0.0075
2, 3, or 4	153 (11.6)	271 (17.3)	−0.1647	251 (15.9)	169 (14.9)	0.0290	137 (14.6)	118 (12.6)	0.0591
Not documented	354 (26.7)	562 (35.9)	−0.1992	487 (31.0)	361 (31.7)	−0.0160	301 (32.1)	292 (31.1)	0.0206
Visceral disease, *n* (%)	444 (33.5)	404 (25.8)	−0.1692	460 (29.3)	337 (29.7)	0.0085	295 (31.4)	293 (31.2)	−0.0046
Bone-only disease, n (%)	519 (39.2)	599 (38.3)	−0.0185	589 (37.5)	440 (38.7)	0.0253	373 (39.7)	403 (42.9)	0.0649
Brain metastases, *n* (%)	26 (2.0)	50 (3.2)	0.0778	26 (1.7)	43 (3.8)	0.1310	18 (1.9)	39 (4.2)	0.1306
Interval from initial breast cancer diagnosis to metastatic breast cancer diagnosis, *n* (%), y									
De novo	541 (40.9)	464 (29.7)	0.2359	530 (33.7)	390 (34.3)	−0.0110	323 (34.4)	323 (34.4)	0.0000
≤1	40 (3.0)	66 (4.2)	−0.0642	74 (4.7)	43 (3.8)	0.0442	34 (3.6)	41 (4.4)	−0.0381
>1–5	191 (14.4)	429 (27.4)	−0.3238	271 (17.2)	288 (25.4)	−0.1992	151 (16.1)	230 (24.5)	−0.2104
>5	551 (41.6)	601 (38.4)	0.0651	696 (44.3)	414 (36.4)	0.1612	430 (45.8)	343 (36.5)	0.1891
Not documented	1 (0.08)	4 (0.3)	−0.0443	1 (0.05)	2 (0.2)	−0.0388	1 (0.11)	2 (0.21)	−0.0267
National Cancer Institute comorbidity index, mean (SD)	0.29 (0.47)	0.39 (0.52)	−0.2096	0.33 (0.57)	0.36 (0.42)	−0.0632	0.31 (0.5)	0.34 (0.5)	−0.0709
Number of metastatic sites, *n* (%)									
1	654 (49.4)	843 (53.9)	−0.0902	793 (50.4)	589 (51.8)	−0.0273	498 (53.0)	526 (56.0)	−0.0599
2	367 (27.7)	291 (18.6)	0.2173	352 (22.4)	261 (22.9)	−0.0136	244 (26.0)	222 (23.6)	0.0543
3	178 (13.4)	133 (8.5)	0.1586	158 (10.1)	129 (11.3)	−0.0413	106 (11.3)	107 (11.4)	−0.0034
4	56 (4.2)	31 (2.0)	0.1298	51 (3.3)	27 (2.4)	0.0501	36 (3.8)	30 (3.2)	0.0347
≥5	33 (2.5)	22 (1.4)	0.0786	33 (2.1)	20 (1.7)	0.0256	19 (2.0)	18 (1.9)	0.0077
Not documented	36 (2.7)	244 (15.6)	−0.4581	186 (11.8)	111 (9.8)	0.0654	36 (3.8)	36 (3.8)	0.0000
Median follow-up duration (IQR), mo	25.0 (13.8–38.3)	23.3 (11.8–42.3)	−0.0049	23.9 (12.8–38.0)	24.5 (12.0–42.9)	−0.0829	23.4 (13.1–37.8)	24.94 (12.4–44.4)	−0.1082

### Overall survival

**Fig. 2:**
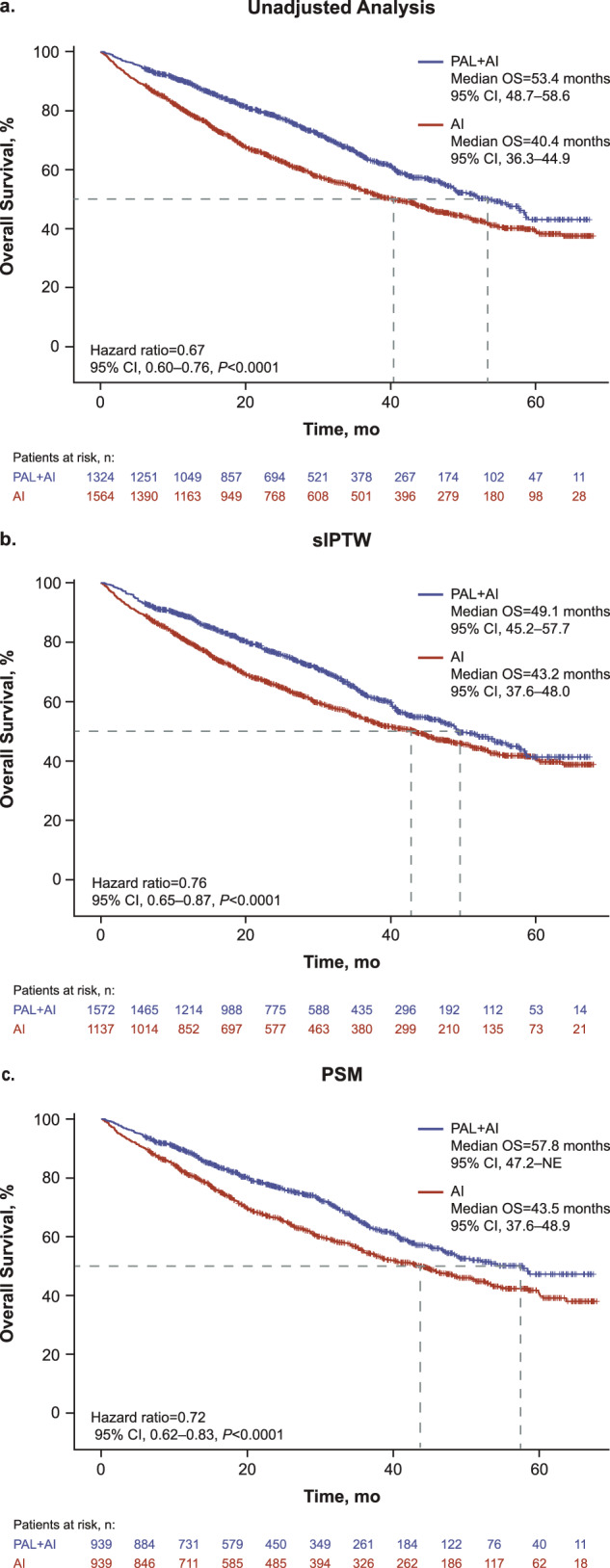
Kaplan–Meier curves of overall survival. AI aromatase inhibitor; NE not estimable; OS overall survival; PAL palbociclib; PSM propensity score matching; sIPTW stabilized inverse probability of treatment weighting. Statistical significance was analyzed by a weighted Cox proportional hazards model.

In the unadjusted analysis of the full cohort (*n* = 2888), median OS was significantly longer among patients in the palbociclib group versus the AI group (53.4 months [95% CI, 48.7–58.6] vs 40.4 months [36.3–44.9]; hazard ratio, 0.67 [0.60–0.76]; *P* < 0.0001; Fig. 2a). After sIPTW, the OS rates at 24, 36, and 48 months were 76.6% versus 65.6%, 62.9% versus 54.4%, and 52.4% versus 46.8% respectively, for palbociclib plus an AI versus the AI alone group. The median OS was 49.1 months (95% CI, 45.2–57.7) in the palbociclib group (*n* = 1572) and 43.2 months (37.6–48.0) in the AI group (*n* = 1137; hazard ratio, 0.76 [0.65–0.87]; *P* < 0.0001; Fig. 2b). After propensity score matching (PSM; sensitivity analysis), median OS was 57.8 months (95% CI, 47.2–not estimable) in the palbociclib group (*n* = 939) and 43.5 months (37.6–48.9) in the AI group (*n* = 939; hazard ratio, 0.72 [0.62–0.83]; *P* < 0.0001; Fig. 2c).

**Fig. 3:**
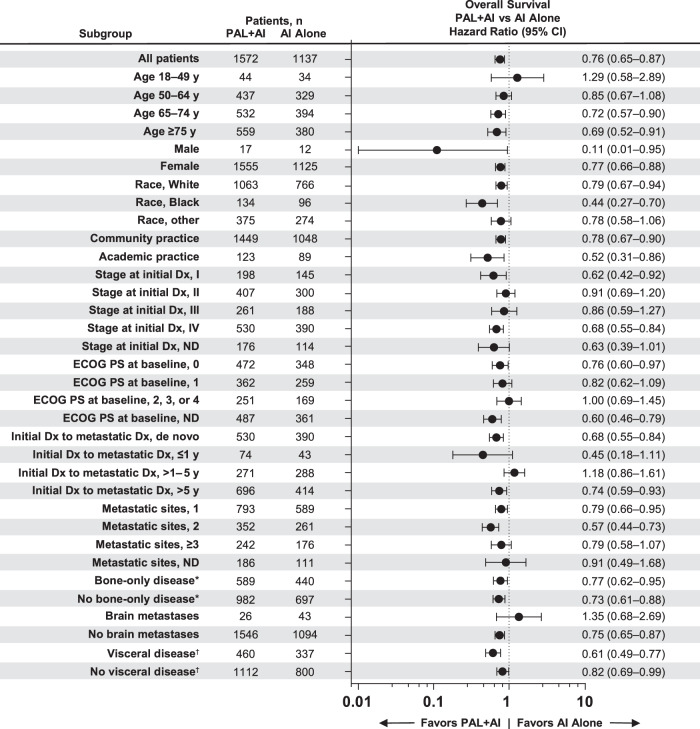
Forest plot of overall survival by subgroup after sIPTW. AI aromatase inhibitor; Dx diagnosis; ECOG PS Eastern Cooperative Oncology Group performance status; ND not documented; PAL palbociclib; sIPTW stabilized inverse probability of treatment weighting. ^*^Bone-only disease was defined as metastatic disease in the bone only. ^†^Visceral disease was defined as metastatic disease in the lung and/or liver; patients could have had other sites of metastases. No visceral disease was defined as no lung or liver metastases.

**Fig. 4:**
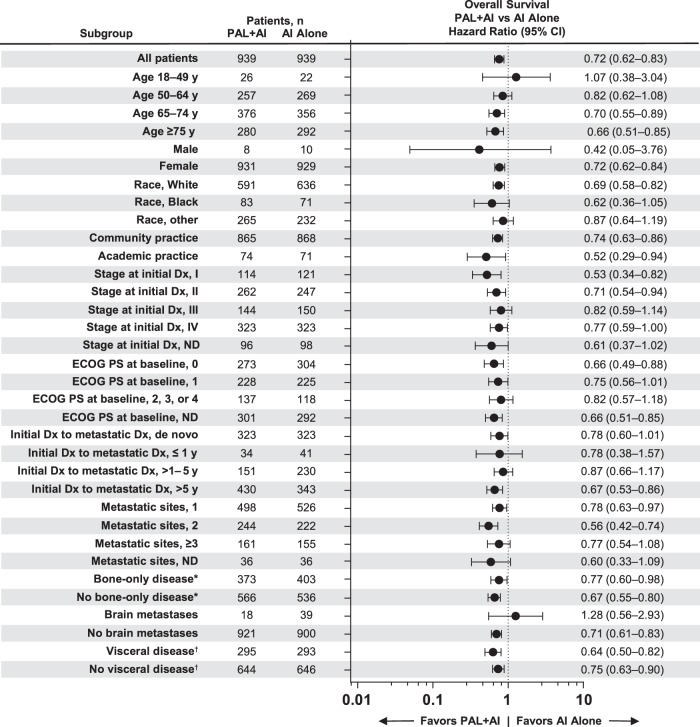
Forest plot of overall survival by subgroup after PSM. AI aromatase inhibitor; Dx diagnosis; ECOG PS Eastern Cooperative Oncology Group performance status; ND not documented; PAL palbociclib; PSM propensity score matching. ^*^Bone-only disease was defined as metastatic disease in the bone only. ^†^Visceral disease was defined as metastatic disease in the lung and/or liver; patients could have had other sites of metastases. No visceral disease was defined as no lung or liver metastases.

A consistent OS benefit with palbociclib plus an AI versus an AI alone was observed across most subgroups examined after sIPTW, regardless of race and among patients with and without visceral disease or bone-only disease (Fig. 3). Similar results were observed in the propensity score matched sensitivity analysis (Fig. 4).

### Real-world progression-free survival

**Fig. 5:**
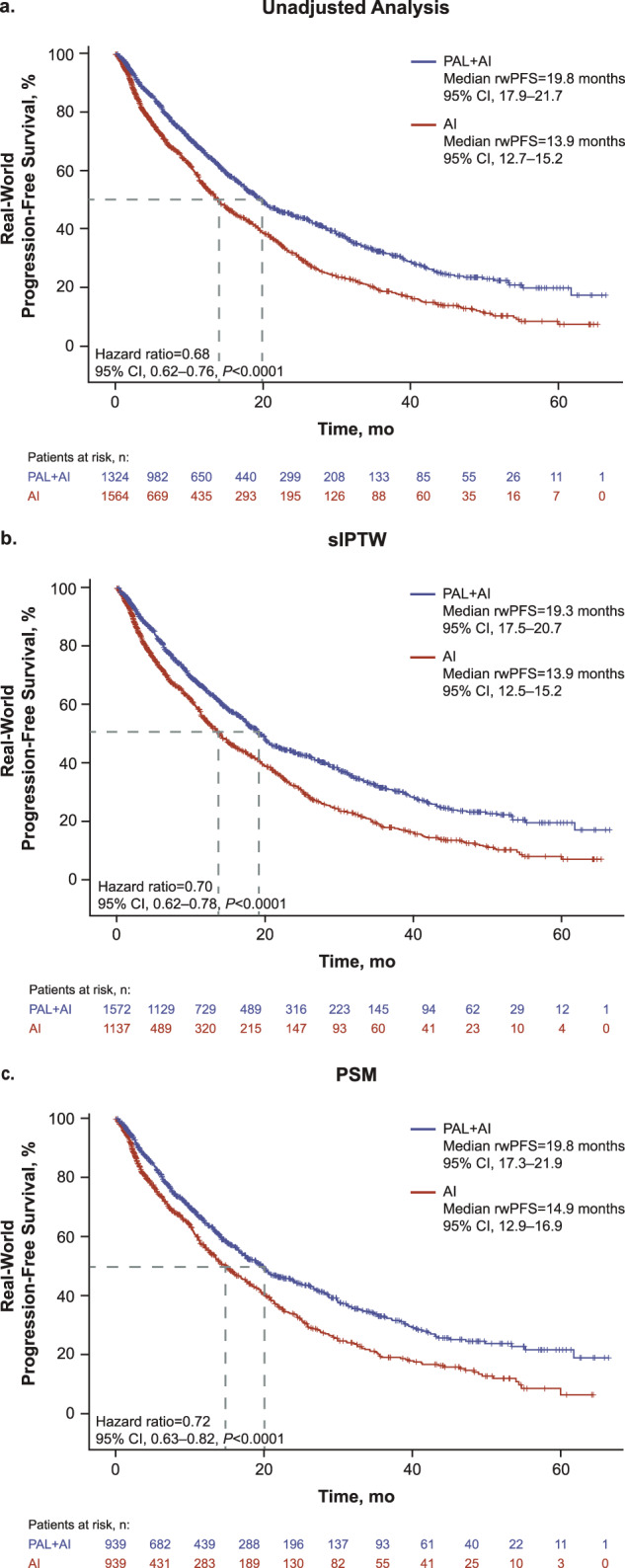
Kaplan–Meier curves of real-world progression-free survival. AI aromatase inhibitor; PAL palbociclib; PSM propensity score matching; rwPFS real-world progression-free survival; sIPTW stabilized inverse probability of treatment weighting. Statistical significance was analyzed by a weighted Cox proportional hazards model.

In the unadjusted analysis of the full cohort, patients in the palbociclib combination group had an associated improvement in median rwPFS that was significantly longer versus patients in the AI group (19.8 months [95% CI, 17.9–21.7] vs 13.9 months [12.7–15.2]; hazard ratio, 0.68 [0.62–0.76]; *P* < 0.0001; Fig. 5a). After sIPTW, median rwPFS was 19.3 months (95% CI, 17.5–20.7) and 13.9 months (12.5–15.2), respectively (hazard ratio, 0.70 [0.62–0.78]; *P* < 0.0001; Fig. 5b). After PSM, median rwPFS was 19.8 months (95% CI, 17.3–21.9) in the palbociclib combination group and 14.9 months (12.9–16.9) in the AI group (hazard ratio, 0.72 [0.63–0.82]; *P* < 0.0001; Fig. 5c).

**Fig. 6:**
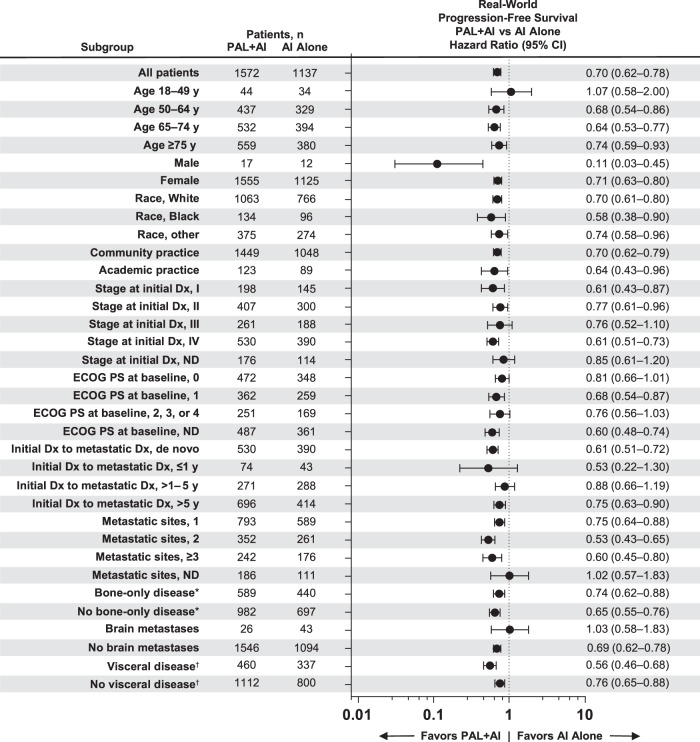
Forest plot of real-world progression-free survival by subgroup after sIPTW. AI aromatase inhibitor; Dx diagnosis; ECOG PS Eastern Cooperative Oncology Group performance status; ND not documented; PAL palbociclib; sIPTW=stabilized inverse probability of treatment weighting. ^*^Bone-only disease was defined as metastatic disease in the bone only. ^†^Visceral disease was defined as metastatic disease in the lung and/or liver; patients could have had other sites of metastases. No visceral disease was defined as no lung or liver metastases.

**Fig. 7:**
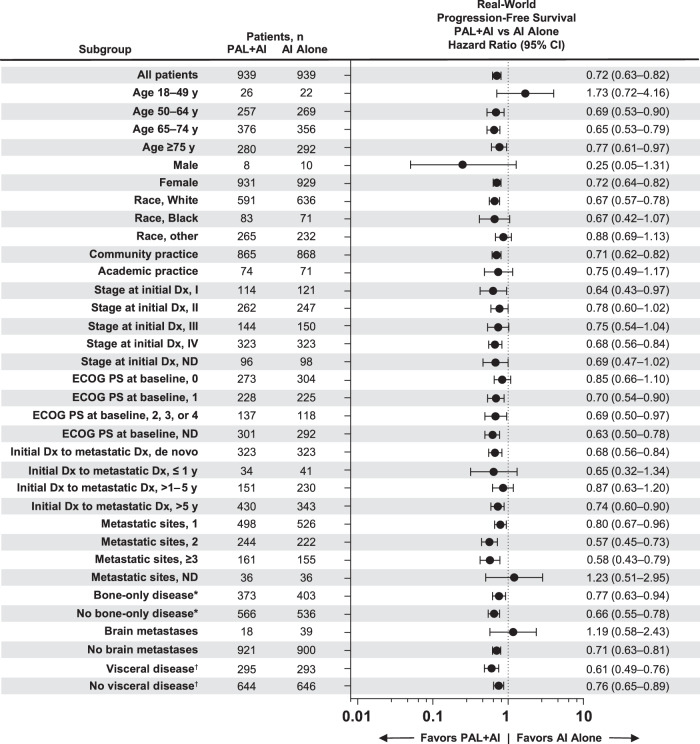
Forest plot of real-world progression-free survival by subgroup after PSM. AI aromatase inhibitor; Dx diagnosis; ECOG PS Eastern Cooperative Oncology Group performance status; ND not documented; PAL palbociclib; PSM propensity score matching. ^*^Bone-only disease was defined as metastatic disease in the bone only. ^†^Visceral disease was defined as metastatic disease in the lung and/or liver; patients could have had other sites of metastases. No visceral disease was defined as no lung or liver metastases.

A consistent rwPFS benefit associated with palbociclib plus an AI versus an AI alone was observed generally across most subgroups examined after sIPTW (Fig. 6). In line with OS results, a rwPFS benefit was associated with the use of palbociclib combination treatment versus an AI alone, regardless of race and among patients with and without visceral disease or bone-only disease. Similar rwPFS subgroup results were observed in the propensity score matched sensitivity analysis (Fig. 7).

### Subsequent treatments

During the follow-up period, 48.9% of patients in the palbociclib combination group and 65.1% in the AI alone group had subsequent treatments. Second-line treatments following first-line palbociclib plus an AI or AI alone after sIPTW analysis are presented in Table 3. Among these patients, 43.1 and 50.5% in the palbociclib combination group and AI group, respectively, received a CDK4/6 inhibitor as second-line treatment, and 21.1 and 15.1% received chemotherapy.

**Table 3:** Subsequent second-line anticancer treatments after stabilized inverse probability of treatment weighting analysis.

Treatments, *n* (%)	Palbociclib + aromatase inhibitor (*n* = 1572)	Aromatase inhibitor alone (*n* = 1137)
First-line treatment only (patients who continued treatment, died, or were censored in the first-line setting)	804 (51.1)	396 (34.8)
Any second-line treatment (patients could have received >1 category of second-line treatment)	768 (48.9)	741 (65.1)
Cyclin-dependent kinase 4/6 inhibitor	331 (43.1)	374 (50.5)
Chemotherapy	162 (21.1)	112 (15.1)
Endocrine therapy alone	154 (20.1)	225 (30.4)
Other anticancer treatment	164 (21.4)	94 (12.7)

## Discussion

Real-world studies are necessary to evaluate the effectiveness of a drug among a heterogeneous population of patients treated in routine clinical practice to inform treatment decisions. Because stringent inclusion and exclusion criteria result in limited diversity in both clinical and demographic characteristics for patients enrolled in randomized clinical trials, the evidence generated may have limited generalizability to actual use in clinical practice. In this retrospective analysis of postmenopausal women and of men with HR+/HER2− MBC in the Flatiron Health Analytic Database, our major finding was that first-line treatment with palbociclib plus an AI was associated with a significantly prolonged OS and rwPFS among all patients and most subgroups analyzed versus treatment with an AI alone. Specifically, an OS and rwPFS benefit with palbociclib plus an AI was observed among de novo MBC patients, patients with and without visceral metastases or bone-only disease, and among subgroups of patients not well represented in breast cancer clinical trials, including Black patients and patients aged ≥75 years. A landmark analysis of OS at 2, 3, and 4 years showed higher OS rates in the palbociclib plus an AI group compared with the AI alone group. Selection of a CDK4/6 inhibitor was also a primary choice as subsequent second-line therapy.

Overall survival is a key endpoint in clinical oncology research. However, an improvement in PFS demonstrated by randomized clinical trials may not result in an improved OS, especially for cancers with long median survival postprogression, possibly due to small sample size, treatment cross over, and the dilution effect of multiple subsequent treatments^22^. Our findings provide real-world effectiveness evidence of CDK4/6i in combination with endocrine treatment versus endocrine treatment alone for HR+/HER2− MBC. It should be noted that recent OS analysis of PALOMA-2 demonstrated that palbociclib plus letrozole numerically prolonged patients’ survival time versus placebo plus letrozole but the OS was not significantly different (HR = 0.96, 95%CI = 0.78–1.18)^10^. Findings from real-world data cannot be directly compared with randomized controlled trials because of differences in study design, inclusion/exclusion criteria, sample sizes, patient characteristics, and data collection. However, there are several potential explanations for the discrepancy in mortality risk reduction between the current real-world study (P-REALITY X) and PALOMA-2. First, OS was a secondary endpoint in PALOMA-2. PALOMA-2 was designed with 90% power to detect a true hazard ratio for the primary endpoint of PFS = 0.69 in favor of the palbociclib arm. The sample size was determined to detect approximately 44% improvement in the primary endpoint of PFS from 9 months for the control arm to 13 months for the palbociclib arm. With OS as a secondary endpoint, the study had 80% power to detect a hazard ratio of 0.74 assuming a median OS of 34 for the control arm improving to 46 months for the palbociclib combination arm (approximately 35% improvement). OS was the primary endpoint in P-REALITY X, which, with 2888 patients (nearly 5 times that of PALOMA-2), would result in greater likelihood of an improvement in OS with at least 80% power to detect a hazard ratio of 0.80. Second, patient characteristics are very different between PALOMA-2 and P-REALITY X. For example, the median age was 61–62 years in PALOMA-2 patients but 70 years in P-REALITY X patients. Most patients in PALOMA-2 were enrolled from academic centers while >90% in P-REALITY X were treated in the community. Third, findings from PALOMA-2 reflect the effect of palbociclib plus endocrine therapy in a small number of patients who met a set of rigorous inclusion and exclusion criteria under closely monitored trial conditions. Findings from P-REALITY X reflect the performance of palbociclib plus endocrine therapy in routine clinical practice and may be more generalizable than those findings from PALOMA-2^23,24^. Furthermore, many factors can have substantial impact on OS, such as comorbid conditions and subsequent therapies. It should be interpreted with caution whether findings between real-world data and randomized controlled trials are consistent or not.

The significant mortality risk reduction with palbociclib plus an AI versus an AI alone in the current real-world analysis (sIPTW, hazard ratio, 0.76 [95% CI, 0.65–0.87]; *P* < 0.0001) is consistent with the OS analysis of MONALEESA-2, a phase 3 study of first-line ribociclib plus letrozole versus placebo plus letrozole in postmenopausal patients with HR+/HER2− ABC (hazard ratio, 0.76 [95% CI, 0.63–0.93]; *P* = 0.004)^25^ and the interim OS analysis of MONARCH-3 (first-line abemaciclib plus AI versus placebo+AI, hazard ratio, 0.75 [95% CI, 0.58–0.97]; *P* = 0.0301^26^. Our findings also support a large single arm observational retrospective medical chart review study in the US and Europe, which demonstrated favorable effectiveness in terms of progression-free and survival rates in patients with HR+/HER2− MBC who received palbociclib with either AI or fulvestrant^27,28^.

Two previous palbociclib comparative analyses conducted using the Flatiron Database demonstrated a significantly associated benefit of palbociclib plus letrozole versus letrozole alone (rwPFS [sIPTW]: 20.0 vs 11.9 months in DeMichele et al; 20.2 vs 16.9 months in Brufsky et al.)^19,20^. However, in both of those analyses median OS was not reached in the palbociclib group. The OS data readout in the current study is a result of a larger sample size (*n* = 2888) than previous analyses (DeMichele et al., *n* = 1430; Brufsky et al., *n* = 1383), as well as longer follow-up time^19,20^. The current study had potential follow-up for ≥6 months from the index date to data cutoff date whereas the previous analyses had potential follow-up for ≥3 months. The current study also included both postmenopausal women and men following the US palbociclib label. Although median OS was not reached in DeMichele et al, the risk of mortality was reduced with palbociclib plus letrozole versus letrozole alone (2-year OS rate, 78.3% vs 68.0%). Overall, the effectiveness findings of the current study (ie, rwPFS and OS) are consistent with those published in DeMichele et al.

In one recent retrospective real-world study of HR+/HER2− MBC (Ha et al), patients who received first-line palbociclib plus an AI versus an AI alone had significantly longer rwPFS, but no significant improvement in median OS was observed in their primary PSM analysis (44.3 vs 40.2 months; hazard ratio, 1.0 [95% CI, 0.8–1.23])^21^. However in a sensitivity IPTW analysis, the hazard ratio for OS was significant (0.79 [95% CI, 0.67–0.93]) and comparable to our primary sIPTW analysis (hazard ratio, 0.76 [95% CI, 0.65–0.87]; *P* < 0.0001). The interpretation of the findings from Ha et al require the consideration of several limitations, including that it was a single academic institution database study and that it included the comparison of patients in the endocrine alone arm from 1997 to 2020 despite the use of a time-to-event analysis; however, palbociclib was not approved until 2015. The Ha et al study also lacked key clinical variables (e.g., ECOG performance status and number of metastatic sites) that may be related to both treatment selection and outcome of interest, thus potentially confounding the findings. Lastly, of note, the mean age of patients was about 50 years in both treatment arms, which was younger than the mean age of patients included in the current study (palbociclib group, 67.1 years; AI alone group, 70.9 years), and is substantially younger than the median age of patients in the US at breast cancer diagnosis (i.e., 62 years)^21,29^.

An expanding body of real-world evidence regarding palbociclib effectiveness adds complementary information to clinical trial data. A recent systematic literature review identified 114 unique real-world studies (inclusive of conference abstracts and posters [*n* = 125] and published journal articles [*n* = 29]) on CDK4/6 inhibitors for HR+/HER2− MBC; among these, the majority of real-world evidence for CDK4/6 inhibitors were in studies of palbociclib (*n* = 79/114)^14^. To date, the current study is the first real-world comparative analysis representing a large and geographically diverse database to report a median overall survival with palbociclib combination therapy for first-line use in HR+/HER2− MBC.

Strengths of this study include the scope and diversity of the Flatiron database. Notably, Flatiron data among patients with MBC have been shown to be comparable to data from the National Cancer Institute’s Surveillance, Epidemiology, and End Results program or Centers for Disease Control’s National Program of Cancer Registries for patients with any stage of breast cancer across sex or geographic location^30^. Additionally, the large sample size (*n* = 2888), long median follow-up, contemporaneous control arm, and prespecified primary and secondary endpoints and sensitivity analysis result in a study with internal validity and valuable effectiveness evidence. Because patients in this observational study were not randomized, differences in baseline and clinical characteristics must be accounted for by using statistical methods to balance patient demographic and clinical characteristics that could confound the analysis (ie, sIPTW and PSM). The significant findings seen in the unadjusted analysis persisted in the sIPTW and PSM analyses. Employing sIPTW as the primary analysis with PSM as the sensitivity analysis confirmed the internal validity of this study. The OS endpoint in the Flatiron Database has been validated against the gold-standard National Death Index and includes external data sources, such as the US Social Security Death Index, obituaries, and commercial death data in addition to health records^31,32^. The rwPFS endpoint measured in this study has also been validated in the Flatiron database^33^. A key strength of this analysis is the inclusion of key variables that can affect treatment selection and survival, including the number of metastatic sites, ECOG performance status, visceral involvement, and the interval from initial breast cancer diagnosis to MBC diagnosis, improving the ability to balance patient cohorts and reduce the risk of confounding^34,35^. The opportunity for real-world evidence to be a component in regulatory decision making continues to evolve, and as standards in real-world study design and transparency in analysis and reporting are adhered to there remains an important opportunity to leverage this data for that purpose^36^. Finally, real-world data may also contain helpful information for international health technology assessment practices that play a role in patient access to innovative treatments^37^.

This real-world study has several potential limitations. First, this study is a retrospective database study of electronic health records, which may have missing or erroneous data entry. In addition, some subgroups analyzed may have insufficient sample size (e.g., younger patients aged <50 years) to identify significant differences in rwPFS and OS outcomes. While sIPTW and PSM were used to balance baseline and clinical patient characteristics, unobserved variables cannot be fully addressed through these methods. Moreover, disease progression was not based on standard criteria (eg, Response Evaluation Criteria in Solid Tumors), but instead was based on the individual treating physician’s clinical assessment or interpretation of radiographic or pathologic results. Lastly, findings presented here may not be generalizable to patient populations not represented in the Flatiron Database.

In conclusion, this is the largest multisite, real-world, comparative effectiveness study to date analyzing CDK4/6 inhibitor combination treatment for HR+/HER2− MBC. Treatment with palbociclib plus an AI was associated with significantly prolonged OS and rwPFS versus an AI alone in a heterogeneous population of postmenopausal women and men with HR+/HER2− MBC. These results were observed across most subgroups. Overall, these data support first-line palbociclib plus an AI as a standard of care for patients with HR+/HER2− MBC.

## Methods

### Study design and data source

This was a retrospective analysis of electronic health records (EHRs) from the Flatiron Health Analysis Database. Flatiron is a longitudinal database that contains de-identified patient data from structured and unstructured EHRs from >280 cancer clinics (~800 sites of care) representing >3 million actively treated patients with cancer in the United States.

For unstructured data abstraction, Flatiron leverages a hybrid approach that pairs ~1500 abstractors, including oncology nurses and tumor registrars, with their proprietary software, Patient Manager, which organizes unstructured documents in predetermined formats. One quality control measure is to have two abstractors complete the same abstraction process for a given patient. In instances when there is abstractor disagreement, the patient data is submitted to an in-house review panel for resolution. As of April 2019, Patient Manager completed computer system–validated activities in line with the US Food and Drug Administration Code of Federal Regulations (Principles in 21 CFR Part 11). Flatiron validated Patient Manager because it is a critical electronic system supporting real-world data handling, with the goal of ensuring that systems are designed and tested appropriately to enable good software practices. To process structured data, Flatiron employs business logic to harmonize and map structured data to a set of universal names and codes for identifying laboratory tests in electronic laboratory report messages or to harmonized drug names. These rules attempt to organize real-world data to facilitate assessment across data points and patient records. The Flatiron Database has been used for multiple real-world studies of treatment patterns and clinical outcomes in breast cancer and other cancers^20,38^.

Data are derived from patients residing in US states, Puerto Rico, and Washington DC. The state field represents the patient’s state of residence. State and territories aligned with the 2-letter convention adopted by the US Postal Service. The state is missing for a small proportion of patients for whom the state of residence was not recorded in the physician’s records. For de-identification reasons, the State is nulled out for all patients at academic institutions and for patients from states with smaller populations (ie, Arkansas, Montana, North Dakota, South Dakota, Vermont, and Wyoming). Any territories outside of the 50 states, District of Columbia, and Puerto Rico are reported in the state field as NULL. This retrospective database analysis was conducted in accordance with the Guidelines for Good Pharmacoepidemiology Practice, Good Practices for Outcomes Research issued by the International Society for Pharmacoeconomics and Outcomes Research, and Good Practices for Real-world Data Studies of Treatment and/or Comparative Effectiveness. As this study is retrospective and non-interventional and uses anonymized data, it is exempt from institutional review board approval and included a waiver of informed consent.

### Study Patients

Patients were selected from the Flatiron Database (Fig. 1). Inclusion criteria included women aged ≥18 years at MBC diagnosis with confirmed HR+/HER2− MBC before or up to 60 days after MBC diagnosis date and were confirmed postmenopausal through chart review. Patients also had a date of first prescription (index date) for palbociclib plus an AI or an AI alone as first-line therapy for MBC between February 3, 2015, and March 31, 2020, and a potential follow-up of 6 to 68 months from the index date to the study cutoff date of September 30, 2020. Exclusion criteria included evidence of prior treatment with CDK4/6 inhibitors, tamoxifen, raloxifene, toremifene, fulvestrant, or chemotherapy in the metastatic setting; first structured activity >90 days after MBC diagnostic date; and lack of relevant unstructured documents in the Flatiron Database for review by the abstraction team.

Clinical characteristics evaluated included visceral disease which was defined as metastatic disease in the lung and/or liver; patients could have had other sites of metastases. No visceral disease was defined as no lung or liver metastases. Bone-only disease was defined as metastatic disease in the bone only. Multiple metastases at the same site were counted as 1 site (e.g., if a patient had 3 bone metastases in the spine, it was considered only 1 site).

### Outcomes

The primary outcome was OS, defined as the number of months from the start of treatment with palbociclib plus an AI or with an AI alone (February 3, 2015) until death. Date of death was derived using a composite of multiple data sources which were benchmarked against the National Death Index^32^. This approach to identify mortality and OS as an endpoint was validated within the Flatiron Database and is important in MBC research because OS estimates can be biased by low sensitivity in mortality surveillance^31^. If patients did not die, they were censored at the study cutoff date (September 30, 2020)^39^.

The secondary outcome was rwPFS, defined as the number of months from the start of treatment with palbociclib plus an AI or with an AI alone to the date of the first documentation of real-world progressive disease or death due to any cause, whichever occurred first^20^. Patients with only one line of therapy who were last known to be alive and progression-free within the follow-up cutoff date were censored at the date of the last clinic note. Patients with more than one line of therapy were censored at the start of second-line treatment. Disease progression was concluded by the treating clinician based on radiology, laboratory evidence, pathology, or clinical assessment. Duration of follow-up was defined as the number of months from start of treatment with palbociclib plus an AI or with an AI alone to death due to any cause or the data cutoff date.

### Statistical analyses

The median OS for an AI alone was assumed to be 40 months. An improvement of 25% to a median OS of 50 months (corresponding to a hazard ratio of 0.80) was considered clinically meaningful. Therefore, 750 OS events were required to have at least 80% power to detect a hazard ratio of 0.80 using a two-sided log-rank test at a significance level of 0.05 based on the exponential distribution assumptions of OS for both cohorts. A total of 1156 deaths occurred by the study cutoff date. Three methods were used and presented for comparative analyses: an unadjusted analysis (without controlling for baseline patient characteristics), the sIPTW method (primary analysis; to control for observed confounders), and the PSM method (sensitivity analysis; to assess the robustness of the sIPTW results). The sIPTW and PSM methods are well-established statistical methodologies that effectively reduce the potential confounding biases in most observational studies due to the lack of randomization. Both methods are based on the propensity score, defined as the probability of assignment to treatment conditional on a set of observed baseline covariates. Propensity scores were generated by a multivariable binomial logistic regression model; variables included in the model were age group, sex, race/ethnicity, practice type, disease stage at initial diagnosis, ECOG performance status, bone disease, visceral disease, interval from initial breast cancer diagnosis to MBC diagnosis, and number of metastatic sites^40–43^. The sIPTW method assigns to each observation a weight, which is calculated as the inverse of its propensity score multiplied by the marginal probability of receiving the given treatment. The PSM method matches observations in one group to observations in the other based on the closeness of their propensity scores. A strength of the sIPTW analysis is that it retains the real-world patient population whereas the PSM analysis reduces the sample size and demonstrates the relative effectiveness in matched patients only. The primary sIPTW analysis was used to balance baseline demographic and clinical characteristics and to adjust for differences in observed potential confounders between the two groups. The balance in important prognostic baseline characteristics was assessed using a standardized differences approach, with a standardized difference of ≥0.10 considered indicative of practical significance^40^. Median survival times and 95% CIs for OS and rwPFS were estimated using the weighted Kaplan–Meier method. The weighted Cox proportional hazards model was used to compute the hazard ratio and the corresponding 95% CI. PSM was conducted as a sensitivity analysis; matches were made using 1:1 nearest neighbor matching without replacement and a caliper of 0.01^40^. All analyses were performed using SAS® version 9.1.4 or higher (SAS Institute, Cary, NC).

## Data Availability

The data that support the findings of this study have been originated by Flatiron Health, Inc. These de-identified data may be made available upon request, and are subject to a license agreement with Flatiron Health; interested researchers should contact <DataAccess@flatiron.com> to determine licensing terms and get the training, data dictionary, validation, and data sets. The Flatiron Health Analytic Database can be contacted at https://flatiron.com/contact/. Interactive visualization of the data presented in this article is available at: https://realworld-data.dimensions.ai/p-reality-x.
